# Tick Infestation Among Stray Dogs of Urban Chișinău, Moldova: Species Distribution and Pathogen Detection

**DOI:** 10.3390/pathogens14121211

**Published:** 2025-11-28

**Authors:** Alexandr Morozov, Anna Victorova, Nadejda Railean, Ion Toderas

**Affiliations:** Institute of Zoology, State University of Moldova, Academie St 1, MD-2028 Chisinau, Moldova

**Keywords:** tick infestation, stray dogs, tick-borne pathogens, urban ecology, Moldova, Chișinău

## Abstract

We investigated tick infestations in stray dogs from Chișinău, the capital of the Republic of Moldova, focusing on tick species distribution, and pathogen infection rates. Ticks were collected from 232 stray dogs across six major urban parks in 2021–2022. A total of 443 ticks were collected, belonging to five species: *Ixodes ricinus* (43.8%), *Dermacentor reticulatus* (35.2%), *Dermacentor marginatus* (20.5%), *Rhipicephalus sanguineus* s.l. and *Haemaphysalis punctata* with only 1 specimen. Notably, 92.5% of ticks were adults, while only 7% were nymphs and <1% were larvae. On average, 44.4% of stray dogs were infested with ticks, with an overall mean intensity of ~4.3 ticks per infested dog. Tick burden varied by location: dogs in large, less-maintained parks carried the most ticks. Tick pathogen screening revealed 24.4% of ticks (108/443) carried at least one pathogen. The most frequently detected were *Babesia* spp. in 12.2% of ticks, *Borrelia burgdorferi* s.l. in 7.4%, *Rickettsia* spp. in 3.4%, *Anaplasma* spp. in 2.5%, and *Ehrlichia* spp. in 0.5%; 4 ticks harbored co-infections. We discuss implications for public health and animal welfare and recommend control measures such as integrated stray-dog management and vegetation maintenance in urban parks.

## 1. Introduction

Ticks are important vectors of numerous zoonotic pathogens, and their presence in urban and peri-urban environments is a growing public health concern [1]. Stray dogs, which often roam freely in cities, can serve as important hosts for ticks and the pathogens they carry [2]. In Chișinău, Moldova, it is estimated that 10,000–20,000 stray dogs inhabit the city’s streets and parks [3,4], potentially acting as maintenance hosts for ticks in the urban ecosystem. Stray dogs are highly vulnerable to tick infestations and to a wide range of tick-borne diseases, including Lyme borreliosis (caused by *Borrelia burgdorferi* s.l.), spotted fever group rickettsioses (SFG *Rickettsia* spp.), and granulocytic anaplasmosis (*Anaplasma* spp.), as well as veterinary pathogens like canine babesiosis (*Babesia canis*). These infections pose a risk not only to animal health but also to humans and domestic pets frequenting urban parks [5]. These free-roaming animals can transport ticks into public spaces and residential areas, increasing the likelihood of human-tick encounters. Indeed, stray dogs have been implicated as reservoirs or amplifying hosts for pathogens causing canine babesiosis, ehrlichiosis, anaplasmosis, spotted fever group rickettsioses (e.g., *Rickettsia raoultii*, *R. slovaca*), and other infections that can also affect humans or domestic pets [6].

Chișinău’s urban landscape includes numerous parks and green areas that provide suitable habitats for tick vectors [7]. The most common tick species in Eastern Europe’s urban and suburban areas are *Ixodes ricinus* and *Dermacentor reticulatus* and *Dermacentor marginatus* [8]. *Ixodes ricinus* is a primary vector of *B. burgdorferi* s.l., the causative agent of Lyme borreliosis and tick-borne encephalitis virus, while *Dermacentor* spp. is known for transmitting *Babesia* spp. [9] and SFG *Rickettsia* spp, the causative agents of tick-borne infections such as tick-borne lymphadenopathy (TIBOLA) and Dermacentor-borne necrosis erythema and lymphadenopathy (DEBONEL) that are commonly transmitted by *Dermacentor* spp. ticks in Europe among other pathogens [10]. These species have a three-host life cycle, but their host preferences differ by life stage. *Ixodes ricinus* larvae and nymphs typically feed on small vertebrates (e.g., rodents, birds), whereas adults feed on larger animals (deer, livestock, dogs, etc.) [11]. Similarly, *Dermacentor* spp. immatures are mostly nidicolous, feeding on small mammals (especially rodents), while the adult ticks seek medium to large-sized mammalian hosts [12].

Stray dogs can thus play an important role as hosts for adult ticks of these species in urban areas where wild large mammals are scarce [13]. In fact, studies have noted that in city parks or peri-urban settlements, dogs may become the dominant hosts maintaining ticks populations [14].

Despite the known presence of ticks and stray dogs in Chișinău [7], there has been a lack of data on the extent of tick infestation in the city’s stray dog population and the associated pathogen carriage. To address this gap, we carried out a survey of ticks collected from stray dogs in various urban parks of Chișinău and tested these ticks for key tick-borne pathogens. We aimed to identify the tick species and life stages infesting stray dogs, quantify the infestation rates, patterns of tick activity, determine the prevalence of infection with pathogens of medical and veterinary importance, and discuss the implications of our findings in the context of urban stray dog management and public health risk. By understanding tick species distribution and pathogen circulation, we can better assess the role of stray dogs in the urban transmission cycle of tick-borne diseases. This knowledge will help identify targeted interventions to reduce risks.

## 2. Materials and Methods

Study area and stray dog examination: The study was conducted in Chișinău, Moldova, focusing on six major urban parks: Valea Trandafirilor, Valea Morilor, Râșcani Park, La Izvor, Florilor, and Alunel. These parks were selected because they represent the largest and most frequently visited green areas in the city, encompassing diverse ecological and management conditions. Together, they capture a broad gradient of park size (from ~0.07 to 2.23 km^2^), vegetation structure (tree and grass cover), and anthropogenic pressure (from intensively maintained recreational zones to semi-natural areas). This diversity allowed us to assess how urban habitat heterogeneity influences tick abundance and pathogen prevalence (Supplementary Table S1). Stray dogs in these parks are unowned, free-roaming dogs that congregate or shelter in the area. We conducted tick collection from stray dogs during 2021–2022. Dogs were approached cautiously and were only handled for inspection if they were docile or accustomed to human contact (many had ear tags indicating prior neutering and vaccination, suggesting they were part of the city’s trap-neuter-release program). Each dog was only inspected with minimal restraint and with humane handling, in line with animal welfare considerations.

Tick collection: To find ticks, the dogs’ coats was palpated and visually examined, focusing on common tick attachment sites such as the head (ears, muzzle), neck, shoulders, limbs, and groin area. Ticks were removed manually using tweezers. Both attached (feeding) ticks and any unattached ticks crawling on the fur were collected. Each tick was placed into a labeled plastic tube. To avoid repeated sampling of the same individual dogs, sampling sessions at each park were structured along routes such that different subpopulations of dogs were encountered on different days (simultaneous tick drag sampling in vegetation confirmed tick presence in the environment, ensuring that ticks collected from dogs were not solely due to a few heavily infested individuals [15]). After removal, ticks were stored in 70% ethanol and later transferred to a laboratory for identification and analysis. Collection took place throughout the active tick season (April–May 2021 and 2022). The geographic location, date, and dog details (estimated age, sex, size, and neuter status if known by ear tag) were recorded for each sampling event (Supplementary Table S1). Overall, 232 unique stray dogs were examined for ticks over the study period (Table 1). Interactive map of the collection’s points with the information of number of dogs and ticks for each point can be found by the link [16]. Collected ticks were identified to species and developmental stage (larva, nymph, adult male or female) using standard morphological keys and descriptions [17]. Identification was performed under a stereomicroscope (Meiji Techno Co., Ltd., Saitama, Japan).

Pathogen detection by PCR: Each tick was subjected to DNA/RNA extraction and screened for the presence of several tick-borne pathogens. Individual ticks were first rinsed in PBS and air-dried to remove ethanol. Nucleic acids were extracted using a commercial kit PREP NA (DNA technology, Moskow, Russia; REF: P-034-N/1EU) following the manufacturer’s protocol, Annex C [18]. Extracts were tested using the multiplex diagnostic real-time PCR kit for tick-borne pathogen targets including *B. burgdorferi* sensu lato, *A. phagocytophilum* and *Ehrlichia chaffeensis/E. muris* (AmpliSens^®^, Moskow, Russia; REF:R-V59); we used the manufacturer’s protocols [19]. The kit includes internal control, positive control and negative control.

For *Rickettsia* spp., which were not covered by a commercial kit, PCRs were performed under conditions previously described by Silaghi et al. [20] and targeted two *Rickettsia* genes: a 380-bp fragment of the gltA gene amplified with primers RpCS.877p/RpCS.1258n, and a 530-bp fragment of the ompA gene amplified with primers Rr190.70p/Rr190.602n [21,22]. All reactions were carried out in a GeneAmp PCR System 9700 thermocycler (Applied Biosystems, Weiterstadt, Germany). Similarly, for *Babesia* spp. we performed a conventional PCR targeting the 18S ribosomal RNA gene. A broad-range primer set (BJ1/BN2) was used, which amplifies a ~400 bp fragment of the 18S rRNA from *Babesia/Theileria* organisms, as previously described [23]. We included positive controls for *Babesia canis* (previously sequenced DNA from a known infected dog blood sample) and *Rickettsia* (previously sequenced DNA from *R. raoultii*-positive tick) to ensure assay performance. Amplicons were analyzed by agarose gel electrophoresis and visualized for the presence of the expected size band. No internal control was used for *Rickettsia* spp. and *Babesia* spp. PCR runs.

Statistical analyses were performed in Microsoft Excel and verified in R (v.4.3.0). Spearman’s rank correlation coefficients (*r*) were used to examine relationships between environmental variables (park area, vegetation structure, grass height, cleaning intensity, mowing frequency) and biological indicators (number of dogs, total ticks, and pathogen-positive ticks). One-way ANOVA performed in Python (SciPy v1.11.1) was used to evaluate whether tick abundance per park differed significantly among parks with different levels of cleaning intensity (0–3), reflecting maintenance frequency and habitat disturbance. The dependent variable was the total number of ticks collected from stray dogs in each park. Because the number of observations varied among parks, the analysis was conducted under an unbalanced design using weighted group means. Homogeneity of variances was verified using Levene’s test. When this assumption was violated, Welch’s ANOVA was additionally applied to confirm the robustness of the results. Differences were considered significant at *p* < 0.05.

## 3. Results

Tick infestation levels in stray dogs: A total of 232 stray dogs were examined in the six parks, of which 105 dogs were found to be infested with one or more ticks. The overall infestation prevalence was 44.3% (Table 1). Infested dogs typically carried a few ticks (median 2 ticks per infested dog), but some had heavy burdens—the maximum observed was 18 ticks on a single dog (in Râșcani Park in late April). Aggregation of ticks on certain host individuals was noted; about 20% of infested dogs accounted for the majority (~60%) of all collected ticks showing a typical negative binomial distribution. In total, 443 ticks were collected from the 105 infested dogs, yielding a mean intensity of ~4.3 ticks per infested dog and a mean abundance of ~1.9 ticks per dog when averaged over all dogs (infested and uninfested). These indices indicate a moderately high level of tick parasitism in the stray dog population.

**Table 1:** The number of examined dogs and the number of ticks collected from them.

Park	N Dogs Checked *	Dogs Infested	N Ticks *
Valea Trandafirilor	43	20	55
Valea Morilor	46	21	98
Râșcani	70	31	175
La Izvor	44	19	78
Florilor	21	9	30
Alunel	8	3	7
***Total***	**232**	**105 (44.3%)**	**443**

* N dogs—number of dogs examined; N ticks—total number of ticks collected.

Five ixodid tick species were recovered (Table 2). The most prevalent were *Ixodes ricinus* and *Dermacentor reticulatus*, which together comprised ~79% of ticks. *I. ricinus* was the single most common species, accounting for 194 ticks (43.8% of the total). *Dermacentor reticulatus* was the second most abundant with 156 ticks (35.2%). *Dermacentor marginatus* was present in significant numbers (91 ticks, 20.5%). Ticks of the *Rhipicephalus sanguineus* s.l. complex were rare—only 1 specimen (an engorged adult female) was found, and only 1 *Haemaphysalis punctata* was collected. Out of 443 ticks, 410 were adults (339 females and 71 males, combined 92.5%). Only 30 nymphs (6.8%) and 3 larvae (<1%) were recorded. (Supplementary Table S1) Tick burdens on dogs varied across the city’s parks (Table 1). Râșcani Park had by far the highest tick counts: 175 ticks (39.5% of total) were collected from 31 infested dogs there, averaging 5.6 ticks per infested dog. Valea Morilor Park had the next highest with 98 ticks from 21 infested dogs. La Izvor Park had 78 ticks from 19 dogs, Valea Trandafirilor 55 ticks from 20 dogs, Florilor 30 ticks from 9 dogs, and Alunel only 7 ticks from 3 dogs. This gradient corresponds with park size, habitat, and stray dog density. Neither dog size nor sex showed a statistically significant effect on tick infestation intensity. Although small dogs and females had slightly higher average tick counts, these trends were not significant (*p* > 0.4).

Pathogen detection in ticks: Out of 443 ticks tested, 109 ticks (24.4%) were positive for at least one pathogen by PCR. The pathogen with the highest prevalence in ticks was *Babesia* spp., detected in 54 ticks (12.2%). Most of these were single infections by *Babesia* (48 ticks), while a few ticks had *Babesia* co-infections with other agents (Table 2).

Correlation analysis revealed that both park size and vegetation structure were strongly associated with the abundance of hosts and vectors. Larger parks and those with greater tree cover showed higher numbers of stray dogs (*r* = 0.82–0.81) and ticks (*r* = 0.68–0.78), as well as higher proportions of pathogen-positive ticks (*r* = 0.81–0.83). In contrast, cleaning intensity and mowing frequency showed strong negative relationships with all biological indicators, particularly for tick abundance (*r* ≈ −0.90). These results highlight the importance of vegetation management and park maintenance intensity in shaping urban tick ecology. One-way ANOVA revealed no significant difference in tick abundance among cleaning intensity categories (F = 0.81, *p* = 0.52). Because Levene’s test indicated unequal variances (*p* = 0.013), results were confirmed using Welch’s ANOVA (F = 0.26, *p* = 0.79), yielding the same conclusion.

## 4. Discussion

Our study provides the first detailed insight into tick infestations of stray dogs in Chișinău, Moldova, revealing a sizable tick burden and the presence of multiple tick-borne pathogens within the urban environment. The results highlight several important points: tick activity on dogs is high in spring; adult ticks vastly outnumber immature ticks on the dogs; the tick fauna is dominated by *Ixodes* and *Dermacentor* species, reflecting infiltration of these ticks from natural foci into the city parks; a substantial fraction of ticks are infected with pathogens, notably *Babesia* and *Borrelia*, among others; stray dogs likely play a significant role in maintaining and dispersing ticks within the city, especially in parks where dog populations and tick habitat overlap; and tick infestation and infection rates vary by location due to environmental and management factors.

Two decades of monitoring reveal a clear chronological shift in the balance between *D. marginatus* and *D. reticulatus* in the Chișinău area. Around twenty years ago, records (which did not distinguish *Dermacentor* species at that time) indicated that the genus *Dermacentor* dominated, comprising approximately 58% of all ticks compared to 41% for *Ixodes* [24]. Ten years ago, vegetation surveys identified *D. marginatus* as the leading species (31.1%) and *D. reticulatus* at 24.5% [25]. Five years later, spring flagging in city parks revealed that *D. reticulatus* had overtaken *D. marginatus*, with proportions of about 20% and 10%, respectively [7]. Our own unpublished vegetation data from the same sites and collection periods show a similar ratio: *D. reticulatus* 22.0%, *D. marginatus* 9.6% [15].

In our current study, the same trend was evident: among ticks collected from stray dogs, *D. reticulatus* accounted for 35.2%, while *D. marginatus* made up 20.5%. Although direct comparison between ticks collected from dogs and from vegetation is imperfect, both *Dermacentor* species share similar host preferences and ambush (questing) behavior, supporting the interpretation of a genuine ecological trend [12]. These observations align with a broader European pattern of *D. reticulatus* expansion in anthropogenic and peri-urban landscapes [11,26], suggesting that local environmental changes such as land-use transformation, increased connectivity of green corridors, and warmer springs favoring early adult activity are driving a shift toward *D. reticulatus* dominance. This compositional change may alter the epidemiological landscape of tick-borne diseases, particularly those linked to *D. reticulatus*, such as canine babesiosis, underscoring the importance of continuous tick surveillance and adaptive management in urban ecosystems [27].

The single *R. sanguineus* s.l. tick was found on a dog in Râșcani park, and the single *H. punctata* in Valea Morilor site. *Rhipicephalus sanguineus* s.l. is reappearing in Moldova after long absence, its presence in Chisinau is registered for the second time in six years after almost 30 years of absence, recently we reported first case of household infestation in near Chisinau area [28]. *Haemaphysalis punctata* is usually associated with livestock or wild ungulates in rural areas; its rare occurrence, according to previous data, accounts for less than 1% of the total tick population in Chișinău. Overall, the diversity of tick species was highest in the larger, whereas small urban park pockets had lower diversity (e.g., park Alunel yielded only *I. ricinus* and *D. reticulatus*).

More than 92% of ticks collected from dogs were adults, while nymphs and larvae were rarely detected. This pattern is most likely related to the collection process rather than a real absence of immature stages. Stray dogs did not allow long or thorough inspection, and sampling had to be quick to avoid stress. Because larvae and nymphs are much smaller and often hidden deep in the fur, they are difficult to notice by touch without fine combing or magnification. In contrast, adult ticks, especially engorged females, are large and easily visible [29]. Thus, the predominance of adults in our samples likely reflects limited inspection time and detection bias rather than true ecological differences.

Stray dogs as tick hosts and public health concerns: Ticks serve as vectors of pathogens affecting both canine and human health. Stray dogs, lacking regular veterinary care, can sustain large numbers of ticks which then can drop off in areas frequented by people. Or an unfed infected *Dermacentor* that rode in on a dog might later crawl onto a park visitor or another pet. Thus, stray dogs facilitate the dispersion of ticks within the urban landscape [30]. In our study, the highest tick counts were in parks (like Râșcani) that also have high stray dog densities—suggesting positive feedback: more dogs → more feeding hosts for ticks → more ticks survive and reproduce → more ticks in environment → more dogs get infested, and so on. This aligns with observations from elsewhere that increases in urban deer or dog populations can boost tick numbers locally [31,32]. In Europe, stray dogs have been recognized as sentinels for tick-borne infections and as possible bridging hosts for zoonoses [33,34,35].

*Babesia* spp. was the most frequent pathogen detected, present in 12% of all examined ticks and in about one-third of *D. reticulatus* females, suggesting an active transmission cycle among stray dogs in Chișinău. Since *D. reticulatus* is known as the main vector of *Babesia canis* in Europe [36], the high positivity among these ticks underlines the importance of monitoring stray dogs as potential reservoirs. Although *B. canis* is not zoonotic, it remains a significant veterinary concern, as infected strays can maintain and spread the parasite to pet dogs visiting the same parks.

*Borrelia burgdorferi* s.l. was identified in 7.4% of ticks. Stray dogs, while not major reservoirs, may contribute to dispersing infected *I. ricinus* ticks and thus serve as practical sentinels for urban tick surveillance.

*Rickettsia* spp. were found in 3.4% of ticks, and *A. phagocytophilum* in 2.5%, both indicating circulation of spotted fever group and granulocytic anaplasmosis agents within the local tick community.

Uneven distribution of ticks among parks—habitat and management factors: Our data show that Râșcani Park had significantly more ticks than other parks, and that larger, less-maintained parks generally had higher tick loads per dog. This pattern likely reflects both habitat suitability and host availability. Parks such as Râșcani and La Izvor, with dense vegetation, marshy zones, and connectivity to open fields or forests, provide ideal conditions for ticks. In contrast, small, manicured parks like Alunel, with frequent mowing and little wildlife, create dry, unfavorable environments. Vegetation management is known to reduce tick density, and our findings support this: Alunel and Florilor, with intensive upkeep, had few ticks, while Râșcani and Trandafirilor, with overgrown areas, had many.

Microclimate and wildlife also play roles [14]. Shaded, humid habitats favor *I. ricinus* survival, whereas open sunny lawns are too dry. Larger parks support more small mammals and birds, sustaining immature ticks. Dog density also appeared to influence tick abundance. For example, Râșcani Park, which had roughly ten times more stray dogs than Alunel, showed correspondingly higher tick numbers. In contrast, Valea Trandafirilor Park, despite offering suitable vegetation and microclimate, had comparatively fewer ticks, possibly due to higher human activity or regular tick-control interventions.

Overall, tick distribution across Chișinău is heterogeneous, with clear hotspots in parks connected to peri-urban natural areas. Urban parks isolated from such corridors had fewer ticks. Identifying these hotspots is essential for targeting control efforts and understanding tick-borne disease risks in the city.

Implications for stray dog management and public health: Chișinău’s stray dog population remains a public health and animal welfare challenge [37]. Integrating vector control into existing stray dog management could mitigate this risk. During neutering or vaccination campaigns, dogs could receive long acting acaricides or oral treatments such as fluralaner, temporarily reducing tick loads and interrupting tick life cycles. Acaricidal collars may also help, if cost and logistics allow. These steps would fit into a broader One Health strategy linking animal and human health.

Public awareness is equally important. City residents should know that ticks are present in parks and practice prevention—using repellents, wearing protective clothing, and checking for ticks after park visits. Promoting tick prevention for owned dogs also reduces the urban tick pool.

Environmental management can further lower tick abundance. Regular mowing and vegetation cleaning in hotspot parks would reduce humidity and tick survival, while avoiding excessive pesticide use [38]. Focused habitat management—keeping grass short, removing leaf litter, and maintaining cleanliness to deter rodents—offers sustainable control. Average grass height also correlated positively with infected ticks (*r* = 0.84), suggesting that dense and poorly maintained vegetation favors both tick survival and pathogen persistence.

Ultimately, improving stray dog welfare through vector management benefits both animals and people. Reducing tick infestations on strays helps limit pathogen circulation in urban ecosystems and should be integrated into municipal public health planning [2].

Comparison with other urban tick studies: In the temperate climatic conditions of Eastern Europe, *Ixodes* and *Dermacentor* are the dominant genera, similar to reports from cities like Warsaw or Berlin where *Ixodes ricinus* is prevalent in parks [39,40]. One study in Kyiv, Ukraine, found *I. ricinus* and *D. reticulatus* commonly infesting dogs and questing in urban and peri-urban vegetation [41,42]. Thus, our results fit within the pattern of an increasing presence of ticks in urban areas across Eastern Europe. The high detection rate of *Babesia* spp. DNA in *Dermacentor reticulatus* ticks collected from dogs is comparable to findings from Poland, where approximately 19% of *D. reticulatus* ticks carried *Babesia* spp. in urban areas [43]. In our study, *Babesia* DNA was identified in about 12% of all tested ticks and was most frequently detected in *D. reticulatus*, although it was also present in a few *I. ricinus* specimens. The prevalence of *Rickettsia* spp. in *Dermacentor* ticks (approximately 9–10%) was lower than reported in Polish studies (38% [43]) but similar to values observed in Germany [44]. These differences might be due to sample size or local enzootic cycles.

## 5. Limitations

Unfortunately, resource constraints did not allow sequencing of positive samples to confirm the detected pathogen species. Currently, there is no convenient and affordable way to send biological samples from Moldova for Sanger sequencing, as international shipment of DNA and tick material requires special export permits and biosafety documentation, which are difficult to obtain and time-consuming. Performing sequencing locally is also prohibitively expensive, even when equipment is available, due to high reagent costs and lack of dedicated funding. We acknowledge the importance of identifying the exact species of *Borrelia* spp., *Babesia* spp., and especially *Rickettsia* spp. detected in our samples; however, this was beyond the scope of the current study. *Rickettsia* spp. are particularly sensitive to this limitation, as genus-level PCR detection does not distinguish between species of differing zoonotic potential. Nevertheless, our results indicate the likely presence of *Rickettsia* from the spotted fever group in the local tick population.

Another limitation was the difficulty in tracking the origin and movement of stray dogs, which may have influenced the spatial distribution of ticks and pathogens between parks.

Recommendations and conclusions: Peak tick season in Chișinău corresponds with late spring; thus, authorities should concentrate tick mitigation and public awareness campaigns in April-May. Signs in parks about tick bite prevention, free tick checks or tick removal stations (as exists in some parks in the EU), and media messages can be timed to spring. Stray dog management programs should integrate ectoparasite control—for instance, including anti-tick treatment in the routine care of captured strays before release, as a standard practice. Collaboration between veterinary services, public health departments, and animal welfare NGOs is essential to implement these measures effectively, in line with One Health principles.

Stray dogs are bridge hosts between the natural tick cycle and the urban human environment. Tackling the issue of tick-borne diseases in cities like Chișinău requires managing this bridge. By reducing tick infestations on stray dogs and managing habitats in parks, we can lower the risk of pathogens spilling over to the human population and also improve the welfare of the dogs (heavy tick loads and tick-borne illnesses contribute to poor health in strays). While Chișinău has already made strides in humane stray dog population control, expanding this to include parasite control will have added benefits.

Stray dogs in Chișinău carry diverse ticks and pathogens, posing a potential public health concern. Our findings offer baseline data to guide local tick control and surveillance efforts.

## Figures and Tables

**Table 2:** Distribution of detected pathogen DNA in ticks collected from dogs across parks of Chișinău.

*Tick Species*	*Park*	*A. phagocytophilum*	*Babesia* spp.	*B. burgdorferi* s.l.	*E. chaffeensis/E. muris*	*Rickettsia* spp.	*Coinfection*	*Neg.*	Total
*Dermacentor* *marginatus*	Florilor	0	0	0	0	0	0	7	7
	La Izvor	0	0	1	0	0	1 (Bor + R)	16	18
	Râșcani	0	5	1	0	2	0	28	36
	Valea Morilor	1	4	0	0	0	0	11	16
	Valea Trandafirilor	1	1	1	0	1	0	10	14
*Dermacentor* *reticulatus*	Alunel	0	0	0	0	0	0	2	2
	Florilor	0	3	0	0	0	0	10	13
	La Izvor	0	6	0	0	1	0	18	25
	Râșcani	2	8	0	0	0	2 (Bab + R) (Bor + A)	48	60
	Valea Morilor	0	4	3	1	2	0	23	33
	Valea Trandafirilor	0	2	1	0	0	0	20	23
*Haemaphysalis punctata*	Valea Morilor	0	0	1	0	0	0	0	1
*Ixodes ricinus*	Alunel	0	0	0	0	0	1 (Bab + Bor)	4	5
	Florilor	1	3	1	0	0	0	5	10
	La Izvor	1	4	3	0	1	1 (Bab + Bor)	24	34
	Râșcani	1	5	12	1	4	2 (Bab + R) (Bab + A)	54	79
	Valea Morilor	1	1	3	0	2	0	41	48
	Valea Trandafirilor	1	2	3	0	0	0	12	18
*Rhipicephalus* *sanguineus s.l.*	Râșcani	0	0	0	0	0	0	1	1
***Total***		**9**	**48**	**30**	**2**	**13**	**7**	**335**	**443**

Bab—*Babesia* spp.; Bor—*Borrelia burgdorferi* s.l. R—*Rickettsia* spp.; A—*A. phagocytophilum*.

## Data Availability

The original contributions presented in this study are included in the article/Supplementary Materials. Further inquiries can be directed to the corresponding author.

## Supplementary Materials

The following supporting information can be downloaded at https://www.mdpi.com/article/10.3390/pathogens14121211/s1, Table S1: Raw data on dogs examined, infestation status, and ticks collected across Chișinău’s urban parks (2021–2022).
